# Accuracy of Computer-Assisted Dynamic Navigation in Implant Placement with a Fully Digital Approach: A Prospective Clinical Trial

**DOI:** 10.3390/jcm10091808

**Published:** 2021-04-21

**Authors:** Cornelia Edelmann, Martin Wetzel, Anne Knipper, Ralph G. Luthardt, Sigmar Schnutenhaus

**Affiliations:** 1 Centre for Dentistry, Dr Schnutenhaus Community Health Centre (CHC) GmbH, 78247 Hilzingen, Germany; edelmann@schnutenhaus.de (C.E.); wetzel@schnutenhaus.de (M.W.); knipper@schnutenhaus.de (A.K.); 2 Department of Dentistry, Clinic for Prosthodontics, Ulm University, 89081 Ulm, Germany; ralph.luthardt@uniklinik.ulm.de

**Keywords:** computer-assisted surgery, computer-aided surgery, dental implants, dynamic navigation, real-time tracking

## Abstract

Background: This prospective clinical study aimed to investigate a possible deviation between the digitally planned implant position and the position achieved using dynamic navigation. The aim of the study was to establish clinical effectiveness and precision of implantation using dynamic navigation. Methods: Twenty consecutive patients received an implant (iSy-Implantat, Camlog, Wimsheim, Germany). One screw implant was placed in one jaw with remaining dentition of at least six teeth. The workflow was fully digital. Digital implant planning was conducted using cone-beam computed tomography (CBCT) and an intraoral scan of the actual condition. Twenty implants were subsequently placed using a dynamic computer-assisted procedure. The clinical situation of the implant position was recorded using an intraoral scan. Using these data, models were produced via 3D printing, and CBCTs of these models were made using laboratory analogs. Deviations of the achieved implant position from the planned position were determined using evaluation software. Results: The evaluation of 20 implants resulted in a mean angle deviation of 2.7° (95% CI 2.2–3.3°). The 3D deviation at the implant shoulder was 1.83 mm (95% CI 1.34–2.33 mm). No significant differences were found for any of the parameters between the implantation in the upper or lower jaw and an open or flapless procedure (*p*-value < 0.05). Conclusion: The clinical trial showed that sufficiently precise implantation was possible with the dynamic navigation system used here. Dynamic navigation can improve the quality of implant positioning. In particular, the procedure allows safe positioning of the implants in minimally invasive procedures, which usually cannot be performed freehand in this form. A clinical benefit and effectiveness can be determined from the results.

## 1. Introduction

Every implant-prosthetic restoration seeks the most natural, functional, and esthetic restoration of the chewing organ after tooth loss [1]. On the one hand, it is essential to protect all neighboring anatomical structures while striving for an implant position and height that favors further prosthetic restoration. Moreover, it is important to optimally use the existing bone of the alveolar process, as a sufficient bony situation around the implant is paramount for the long-term success of the implant [2,3]. Other key aspects, such as distances between implants or between implants and their neighboring teeth, the [4] soft tissue situation, or the [5] position of the cement gap, must [6] also be considered. With the help of prosthetic-oriented planning, an optimal and predictable result can be achieved [7]. A study with a follow-up period of 1 year showed that the computer-assisted procedures had no influence on the implant survival rate compared to the conventional procedures [8].

Potentially serious surgical complications, such as injury to the anatomical structures (e.g., nerves, vessels, or the maxillary sinus), can be reduced by computer-assisted procedures [9]. It is an advantageous treatment option for edentulous patients if a suitable procedure without the formation of a mucoperiosteal flap (i.e., flapless) is available [10]. In a systematic review, Gargallo-Albiol et al. presented that a flapless procedure significantly increased patient comfort. Thus, patients experienced less post-operative pain and less swelling in the surgical area. Furthermore, fewer painkillers were used, and the operation was shortened [11].

Three-dimensional (3D) planning forms the basis for computer-assisted implantations. In this case, the anatomical situation of the implant region is merged with the digitized prosthetic planning goal using 3D computed tomography (CT) or cone-beam computed tomography (CBCT) data [1]. Digital implant planning can be implemented using computer-assisted procedures [12]. Static navigation using drilling jigs is an established procedure [13]. Clinically sufficient accuracy and prosthetically predictable results can be achieved using this procedure [11,14]. Numerous studies on guided implant placement have attempted to determine the factors that affect the accuracy of the procedure, including different manufacturing methods or different materials used for drilling jigs [15,16,17]. Different drilling sleeves [18,19,20], as well as the intraoral positioning and fixation of the jigs [21], can influence accuracy. However, application errors in the implementation seem to generate greater inaccuracies than the method per se [22].

In addition to static processes, there are also dynamic computer-assisted processes for transferring 3D planning to clinical patient situations [1]. During the implant bed preparation and implant insertion, the surgeon can navigate the 3D position of the instruments in real-time on a screen according to the planning [23]. This is allowed by optical tracking systems that recognize defined reference [24] markers. Various preclinical studies are available for these systems [25,26,27].

In recent years, dynamic navigation has been increasingly used clinically, owing to the further development of computer technology and associated computer-aided processes [1]. Dynamic navigation is advantageous because it can use any implant system, owing to open-source systems; moreover, there are no downsides connected to laying a static jig, especially with a flapless procedure [1]. Compared to fully-guided static navigation, surgeons may consider intraoperative, situation-related changes [11]. The overheating of drills due to insufficient coolant access, such as those arising from closed drilling jigs, can be avoided [28]. The implant bed can also be prepared with a small mouth opening or in regions that are far distal in which the vertical space is limited [29]. However, due to a pronounced learning curve and the complexity of the surgical procedure, practitioners and the entire surgical team must familiarize themselves with it [30]. Dynamic navigation assumes that the position of the instruments can be recognized by a reference body and are assigned during virtual planning. The placement of markers differed significantly according to the system. A systematic review of clinical studies on the accuracy of dynamic computer-aided navigation showed comparable results to those of static navigation. However, individual dynamic navigation systems show great heterogeneity, which must be considered [31]. We may assume that further development of surgical procedures through virtual and augmented reality technologies will increase the quality of implantological care [32]. In the present study, the system was used for the intraoral securing of the reference marker. This clinical study sought to investigate the accuracy of implantation using dynamic navigation and to determine the indications of possible influencing factors.

## 2. Materials and Methods

### 2.1. Study Design

In this controlled prospective clinical study, 20 screw implants (iSy, Camlog, Wimsheim, Germany) were placed in one jaw of patients with a remaining dentition of at least 6 teeth. The deviation of the planned implant position from the position achieved using dynamic navigation was investigated. The study inclusion and exclusion criteria were as follows:

Inclusion criteria:

- Submission of written informed consent;
- Restoration of at least one missing tooth using an implant;
- At least six residual teeth in the affected jaw.

Exclusion criteria:

- People under 18 years of age or people without legal capacity;
- The use of a reference marker for implant placement is not possible (restricted mouth opening);
- Necessary additional augmentation requirements;
- Heavy smoker (>10 cigarettes/day);
- Immediate implant placements;
- Intake of bisphosphonates;
- Pregnant women;
- Alcohol and/or drug abuse;
- Patients with infectious diseases, such as hepatitis or AIDS;
- Poorly controlled diabetes mellitus.

Patients were recruited following registration of the study with the German Clinical Trials Register (DRKS-ID: DRKS000023687) and approval by the ethics committee of the State Medical Association of Baden-Württemberg, Germany (Application No.: F2020-113-z). Implants were placed between November 2020 and March 2021. Patient data were collected in the practice of PD Dr. Schnutenhaus in Hilzingen (cooperating partner of the Clinic for Dental Prosthetics, Ulm University Hospital). In cases where multiple implants were to be placed in a patient, a test implant was specified to maintain independent values. After planning the position and number of implants, the test implant was determined preoperatively by randomization using the randomization function in Excel (Microsoft Corporation, Redmond, WA, USA).

### 2.2. Implant Planning

Implant planning was conducted using the implant planning software coDiagnostiX Version 9.11 (Dental Wings GmbH, Chemnitz, Germany). As the first step, a CBCT image (PaX-i3D Green nxt, Orangedental, Biberach, Germany) and intraoral digitization of the jaw (Trios 3, 3Shape, Copenhagen, Denmark) were created. For planning purposes, both datasets were loaded into the planning software and digitally assigned to one another. For implant planning, a virtual set-up was created according to the prosthetic principles. All planning was performed by a practitioner (SiS).

### 2.3. Reference Marker

**Figure 1:**
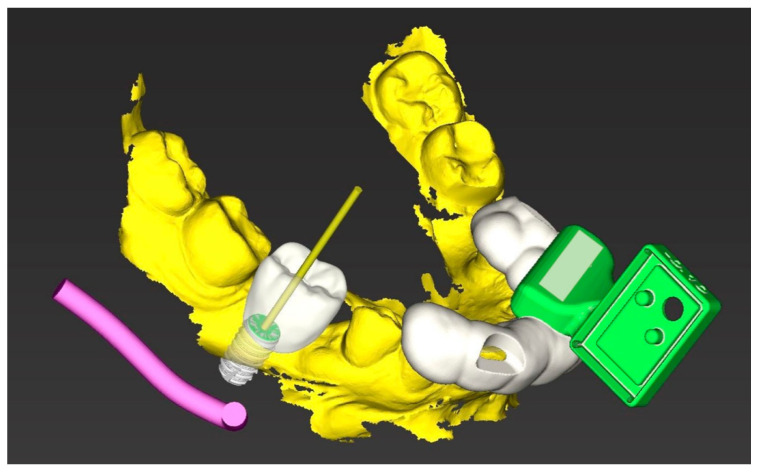
The model (yellow) with implant planning and design of the marker tray (white) with the holder (green).

After completion of the planning, the 3D object was loaded into the planning software. This 3D object represents the basis of the holder as a reference marker. The object was oriented in such a way that starting from the implant region, it was placed on the contralateral premolar/molar region above the row of teeth. The designer for drilling jigs was subsequently used to design a holder that could be securely attached to the teeth in the contralateral quadrant, where the marker was fixed before surgery (Figure 1).

The design data of the marker jigs were sent to an in-house dental laboratory. All of the marker jigs were printed by a dental technician using a 3D printer (Straumann CARES P20, Straumann AG, Basel, Switzerland). The jigs were cleaned and post-cured according to the manufacturer’s instructions. The printed holder was sterilized with a fixed marker and positioned on the row of the teeth during surgery.

Digital planning was passed on to the navigation system when the files were output on a USB stick. The data of the respective CBCT, planning, and positioning of the marker were converted into a data record that could be read by the navigation system. This conversion was a program function of coDiagnostiX planning software.

**Figure 2:**
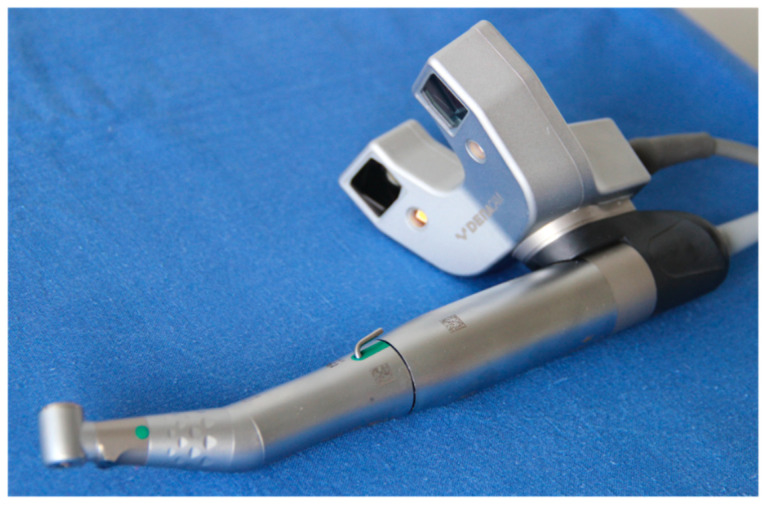
The surgical handpiece with the camera attached.

**Figure 3:**
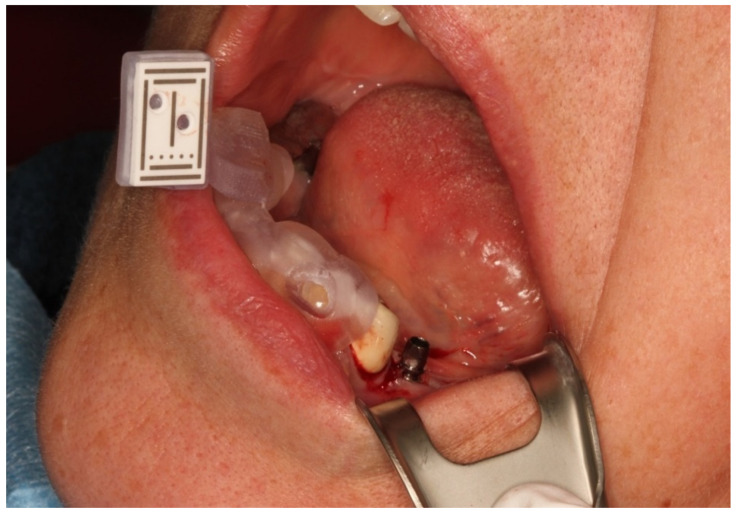
Patient situation with the fixed reference marker region 43/44 and implant insertion region 35.

**Figure 4:**
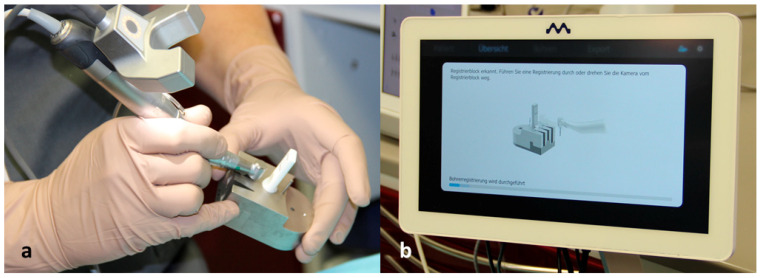
(**a**) Registration of the respective drill. (**b**) The registration progress is displayed on the screen.

**Figure 5:**
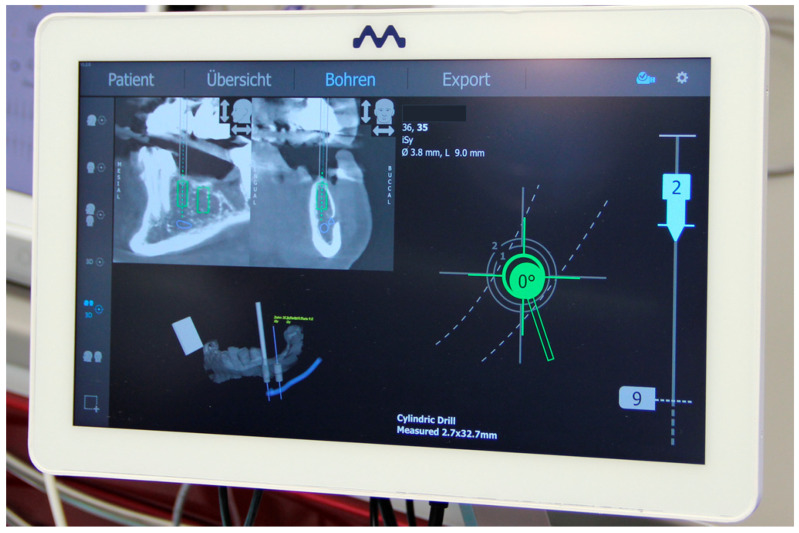
Real-time representation of the drill position (the position, angle, and depth) as well as a sectional view of the planned implant position in the CBCT.

The data were transferred to the DENACAM navigation system (Mininavident AG, Liestal, Switzerland). The DENACAM system works with a camera attached to the surgical handpiece (Figure 2). A marker was placed in the mouth as the reference structure (Figure 3). Each drill, including the implant itself, was automatically registered using a registration instrument (Figure 4) before being used. This step was necessary to provide precise information to the system on the length and diameter of the drill being used. This was guided interactively using a 3D display on a screen that was clearly visible to the surgeon. The position, angle, and depth were displayed in real-time (Figure 5).

**Figure 6:**
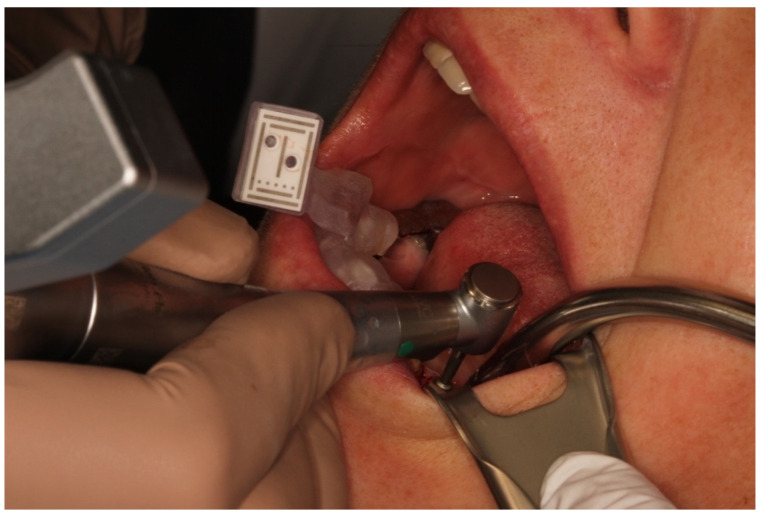
The process of the implantation.

Implantation was conducted according to the implant manufacturer’s drill protocol. All implantations were performed by an experienced dentist (SiS) (Figure 6). All of the steps were conducted mechanically using a contra-angle handpiece. The end position of the implant was determined using the DENACAM system’s display. Neither the visual control nor the readjustment of the implant position was performed.

### 2.4. Registration of the Implant Position

**Figure 7:**
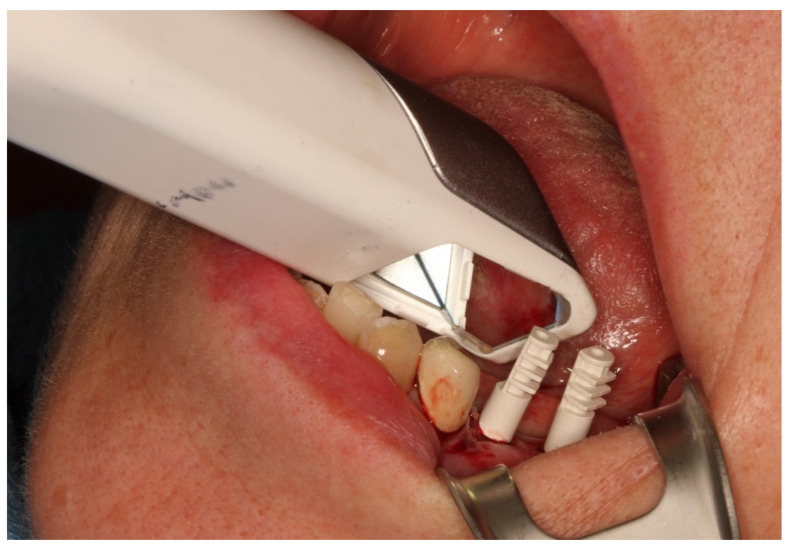
Digital impression of the implants in regions 35 and 36.

After implantation, multifunctional caps were placed on the implants as a scan body to register the implant position. The clinical situation was optically digitized using an intraoral scanner (Trios 3, 3Shape, Copenhagen, Denmark) (Figure 7).

The models were then designed using an automatic software process (3Shape, Copenhagen, Denmark) and produced with the Straumann CARES P20 printer. A laboratory analog (iSy-Implantatanalog, Camlog, Wimsheim, Germany) was incorporated into these models to transfer the clinical implant position to the model situation. A CBCT image of the models was produced and saved in Digital Imaging and Communications in Medicine (DICOM) format. These datasets were integrated into the original digital plans for the evaluation.

**Figure 8:**
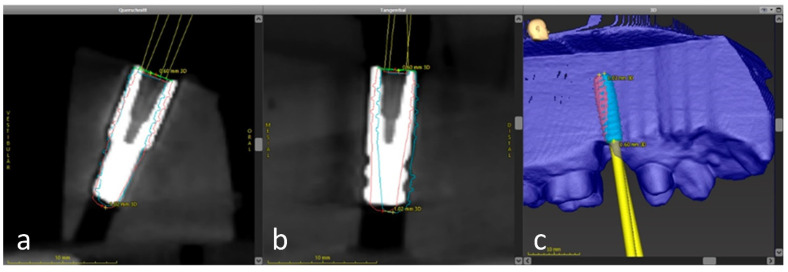
Overlay of the planned (cyan) and the achieved implant position (pink) using the treatment evaluation program function in the coDiagnostiX software. (**a**) sagital direction (**b**) transversal direction (**c**) 3-D visualization

The automated surface best-fit matching with the iterative closest point algorithm in the treatment evaluation mode, coDiagnostix software version 9.11, was used to overlay the preoperative CBCT with the postoperative CBCT scans of the model (Figure 8). First, the model dataset was superimposed on the CBCT data set based on the remaining hard tooth substance. Subsequently, an implant cylinder was manually aligned in all cutting planes to the laboratory analog that corresponded to the clinically achieved implant position. The overlay and evaluation were carried out by a dentist (MW) who was not involved in either planning or implant placement.

### 2.5. Analysis of the Implant Position

The metric analysis included the following measurements:

3D deviation: The 3D deviation of the midpoints between the implant planning and the clinically achieved implant position, measured at the implant shoulder and apex (corresponding to the Euclidean distance).

Apico-coronal deviation (height difference): The vertical offset in the vertical direction, measured at the center of the implant shoulder.

Axis deviation: The angular deviation of the implant axes from planning and clinically achieved implant position.

Two-dimensional deviation in the mesiodistal and oro-vestibular directions was measured at the implant shoulder and axis.

The measurement method was based on the principle of Tahmaseb et al. [21] to enable better comparability with the current and future studies.

### 2.6. Statistical Analysis

The mean values, standard deviations, 95% confidence intervals (CIs), and minimum and maximum values are provided for the variables. After testing for normal distribution, statistical testing was performed. For normally distributed values, a t-test was performed for the mean values of independent random samples to compare the planned and achieved implant positions. If the values were not normally distributed, the Mann–Whitney U test was performed.

Statistical significance was set at *p* < 0.05. All statistical analyses were conducted using SPSS^®^ Statistics version 27 (IBM Corp. Released 2020, Armonk, NY, USA).

## 3. Results

### 3.1. Description of the Study Population

Of the 20 patients, 13 were women and seven were men. The mean age was 55.6 years (range, 33–84 years). The distributions by sex, surgical procedure, and jaw, are shown in Table 1. The length and diameter of the implants are listed in Table 2.

### 3.2. Evaluation of the Implantations

A total of 20 implants were evaluated. The implantations showed a mean deviation of 1.83 mm (95% CI, 1.34–2.33 mm) in the 3D deviation at the implant shoulder, and a mean 3D deviation of 1.95 mm (95% CI, 1.40–2.50 mm) at the implant apex. The mean angular deviation was 2.7° (95% CI, 2.2°–3.3°). The data for all the measured values are listed in Table 3.

**Figure 9:**
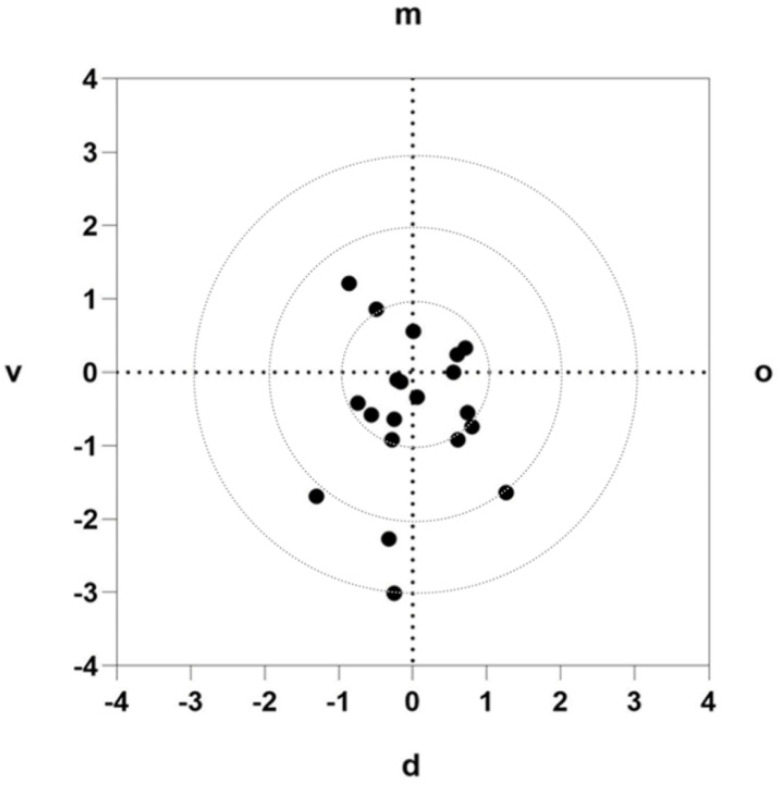
Two-dimensional deviation at the implant base: representation of the exit points in the oral (o), vestibular (v), mesial (m), and distal (d) directions.

The two-dimensional deviation at the implant base is shown in Figure 9. Of the 20 implants, 70% were further distal at the implant exit point, 55% were further vestibular, and 90% were further coronal than planned.

Data were analyzed relative to the insertion in the upper and lower jaws, as well as to the type of surgical procedure (formation of a mucoperiosteal flap = flap or flapless). No significant differences were observed between the groups (Table 4). Neither the jaw nor the surgical approach had any effect on the results. The gender of the patients had likewise no statistically significant influence on the accuracy.

## 4. Discussion

The goal of implant prosthetic restoration is the functional and esthetic restoration of the missing teeth. A prosthetically based optimal implant position is the basis for perfect prosthetic restoration. Various computer-assisted procedures have been established for dental implants. In particular, these are static procedures with the aid of drilling templates. Dynamic procedures are increasingly being used and investigated in clinical studies. These dynamic procedures should have comparable accuracy to the well-established static procedures.

The results of the present study had a mean deviation of 1.83 mm (95% CI, 1.34–2.33 mm) at the coronal end of the implant and 1.95 mm (95% CI, 1.39–2.50 mm) at the implant apex. The mean angular deviation was 2.7° (95% CI, 2.2°–3.3°).

The small sample size of 20 patients was one of the major limitations of the present study. Furthermore, only one implant system was examined, and all of the implants were placed by an experienced practitioner. Further studies examining different implant systems and the influence of the practitioner are necessary. The position and fit of the holder are potential sources of error that can negatively affect the accuracy. The position of the holder, like that of a surgical template, must be constantly checked during the procedure in static navigation [13]. The evaluation process was based on the superposition of two 3D data sets: the CBCT scan of the patient situation and the printed model with a laboratory analog. Every single step in the data collection, as well as the resolution of the scan, print, and CBCT data could cause inaccuracies. Individual components should operate as precisely as possible so that the sum of the errors at the end could result in a clinically tolerable deviation in the implant position [33]. An intraoral scan was performed to register the implant position. Thus, the intraoral scan was significantly more precise than the CBCT on which the planning was based upon.

To date, few clinical studies on current methods have investigated the accuracy of implant positions in dynamic navigation [34,35,36,37,38,39,40,41,42]. Previously published data on the accuracy of dynamic navigation were analyzed in three systematic reviews. On average, the coronal 3D deviation at the implant shoulder was between 1.00 mm (95% CI 0.83, 1.16 mm) and 1.11 mm (95% CI 0.96, 1.26 mm) [31,43,44]. The implant exit point is particularly important for prosthetically predictable results. It has a decisive influence on the esthetic results [45]. Thus, the values of this study are at the upper end of previous studies on the accuracy of dynamic navigation. The consideration of apical deviations was particularly relevant for maintaining the safety distances. On average, the 3D offset at the implant tip was 1.95 mm (95% CI, 1.39–2.50 mm). A safety distance of at least 2 mm, as specified for static navigation, must be observed [21,43].

The evaluation of the angular deviation in the present study showed a mean deviation of 2.7° (95% CI, 2.2°–3.3°). These values showed a similar precision compared to the majority of studies published to date [31]. The angular deviations reported in these studies were between 3.68° (95% CI, 3.61°–3.74°) [43] and 4.22° (95% CI, 2.74°–5.68°) [44]. Without the use of individual abutments, inclined implant axes could impair the correct design of proximal contacts.

Linear deviations in the present study were 0.93 mm (95% CI, 0.58–1.29 mm) in the mesiodistal direction and 0.48 mm (95% CI, 0.30–0.65 mm) in the oro-vestibular direction. The accuracy shown here was consistent with that described in the literature on dynamic navigation. Therefore, the co-factor of the height offset in the 3D deviation had a decisive influence on our study. The final implant position was reached using a display in the navigation system. No subsequent corrections were made under visual control. As a rule, the height of the implant in relation to the crestal bone could easily be checked and corrected intraoperatively, provided that implant planning had taken into account a sufficient safety distance in the axial direction. The height offset and the resulting larger 3D deviations could also be due to the macro design of the implant system used. Implants with a strongly conical implant design also showed higher deviations in static navigation [46].

The accuracy analysis by jaw and type of the surgical procedure should indicate whether these influenced the accuracy between the planned and achieved implant positions. Values in the oro-vestibular direction at the implant base in the upper jaw were significantly worse. No other parameters showed any significant differences. The surgical approach did not seem to affect the accuracy. Due to the small number of patients, results could be considered merely informative; further studies are necessary to verify this tendency.

Various factors that influence static navigation have been investigated. In addition to the template design and the storage and fixation of the template itself, surgical access (open vs. closed) can also influence the accuracy [47]. Another factor with a significant influence on accuracy is the type of implant [48]. In a retrospective study by Wu et al., no significant differences between the dynamic and static navigations were found at the coronal or apical endpoints or in the angular deviation when evaluating a total of 95 implants [41]. Yimari et al. also reported a similar result in a randomized clinical trial [42]. In an in vitro study by Mediavilla-Guzmán et al., only significantly different values in favor of static template-guided implantation for angular deviation were reported. The static group averaged at 2.95° ± 1.48°, in contrast to the dynamic implantation with 4.00° ± 1.41° [49]. These data are in contrast with another in vitro study where a dynamic method was compared with a static method, and significant differences in all parameters were shown in favor of the static method [50].

The results of in vitro investigations could be transferred to the clinically achievable accuracy of the system presented here. In a systematic review by Wei et al., no significant differences between in vitro and clinical studies were observed [44]. In the clinical implementation of dynamic navigation on the patient, possible influencing factors, such as the opening of the mouth, movements of the patient, or the restricted view of the operating field, did not seem to cause any deterioration in the results.

In contrast to static navigation, there are still few published clinical studies on dynamic navigation [34,35,36,37,38,39,40,42]. The comparability of these studies is difficult because of heterogeneous co-factors, such as the use of different dynamic navigation systems, different implant planning programs, and different implants. The 3D deviations at the implant exit point are between 0.67 ± 0.29 mm and 1.37 ± 0.55 mm. The mean values of the angular deviations are between 2.26° ± 1.62° and 6.46° ± 3.95°.

The dynamic method values lie in a range similar to that of static navigation [13,51,52]. However, the studies presented (clinical and in vitro) showed high scatter in terms of accuracy. Further investigations should indicate whether this is mainly due to the dynamic navigation systems used. Another problem is that each navigation system uses different reference structures. Therefore, the respective studies are hardly comparable. Additionally, in most cases, only one clinical study of each workgroup is available. The IRIS-100 system (EPED Inc., Kaohsiung City, Taiwan) has already been used in two independent clinical studies. The results showed a comparable precision with mean coronal deviations of 1.24 ± 0.39 mm vs. 1.05 ± 0.44 mm and an angular deviation of 3.78° ± 1.84° vs. 3.06° ± 1.37° [37,42]. Wu et al. did not find any significant differences arising from operating surgeons and their experience levels [41]. These results suggest that the values are also somewhat robust when used by different surgeons. The results of our investigation were slightly worse than those of static navigation. This might be because the preparation was conducted virtually freehand without the guidance of a sleeve system. In addition to accuracy, the feasibility of this method in everyday clinical practice must be verified in further examinations.

## 5. Conclusions

The present clinical investigation showed that the dynamic navigation system used here could provide a sufficiently precise implementation of the planned implant position. The accuracy was slightly lower compared to data from the literature on the results that can be achieved with static navigation methods. Predictable accuracy can be achieved with dynamic navigation. A clinical benefit and effectiveness can be determined from the results.

The workflow could be fully digital. When classifying the accuracy achieved here, we could assume a dependency on the system used. The success of the dynamically navigated implantation required intensive training because of the non-trivial surgical implementation. Further clinical studies must examine and assess the methods of dynamic navigation compared with freehand implantation and static navigation.

## Figures and Tables

**Table 1:** Frequency distribution of evaluated implants (*n* = 20) relative to the gender, jaw, and surgical procedure.

	Male	Female	Upper Jaw	Lower Jaw	Flap	Flapless
Frequencies	7	13	4	16	10	10
Percent	35%	65%	20%	80%	50%	50%

**Table 2:** Distribution of implant lengths and diameters.

Lengths/Diameters (mm)	3.8	4.4	5.0
9	2	9	3
11	1	3	1
13	1	0	0

**Table 3:** Mean, standard deviation, minimum and maximum deviation at implant shoulder, and apex and axial deviation.

		Mean (SD)	95% CI	Minimum	Maximum
Angle	Deviation	2.7 (0.26)	2.2–3.36	1.1	5.9
Shoulder	3D Deviation	1.83 (0.24)	1.34–2.33	0.53	4.14
	Mesiodistal Deviation	0.93 (0.17)	0.58–1.29	0.00	3.01
	Oro-vestibular Deviation	0.48 (0.08)	0.30–0.65	0.01	1.30
	Apico-coronal Deviation	1.31 (0.22)	0.85–1.78	0.02	3.68
Apex	3D Deviation	1.95 (0.28)	1.39–2.50	0.51	4.37
	Mesiodistal Deviation	1.07 (0.22)	0.62–1.52	0.10	3.88
	Oro-vestibular Deviation	0.57 (0.08)	0.40–0.74	0.10	1.58
	Apico-coronal Deviation	1.30 (0.22)	0.84–1.76	0.03	3.68

SD, standard deviation; CI, confidence interval.

**Table 4:** Deviations between planned and achieved implant positions.

		Lower Jaw *n* = 16			Upper Jaw *n* = 4			*p*-Value	Open Procedure *n* = 10			Flapless *n* = 10			*p*-Value
		Mean (SD)	95% CI	Min.–Max.	Mean (SD)	95% CI	Min.–Max.		Mean (SD)	95% CI	Min.–Max.	Mean (SD)	95% CI	Min.–Max.	
Shoulder	3D	1.77 (0.24)	1.25–2.29	0.53–3.84	2.08 (0.74)	0.28–4.44	0.82–4.14	0.617	1.78 (0.39)	0.91–2.67	0.53–3.84	1.88 (0.29)	1.22–2.54	0.82–4.14	0.162
	Mesiodistal	0.93 (0.19)	0.52–1.34	0.00–3.01	0.95 (0.42)	0.37–2.27	0.13–1.69	0.958	1.02 (0.30)	0.34–1.70	0.00–3.01	0.84 (0.17)	0.45–1.23	0 13–1.69	0.179
	Oro-vestibular	0.42 (0.06)	0.29–0.56	0.01–0.86	0.70 (0.34)	0.38–1.77	0.06–1.30	0.195	0.39 (0.09)	0.19–0.58	0.01–0.80	0.57 (0.14)	0.25–0.88	0.06–1.30	0.184
	Apico-coronal	1.26 (0.23)	0.77–1.75	0.02–3.68	1.53 (0.68)	1.26–2.45	0.64–3.55	0.641	1.17 (0.35)	0.38–1.96	0.02–3.68	1.46 (0.69)	0.83–2.09	0.64–3.55	0.532
Apex	3D	1.86 (0.28)	1.26–2.45	0.51–4.06	2.30 (0.76)	0.11–4.72	1.10–4.37	0.512	1.89 (0.45)	0.87–2.90	0.51–4.06	2.00 (0.30)	1.32–2.69	1.10–4.37	0.052
	Mesiodistal	1.03 (0.24)	0.51–1.55	0.03–3.88	1.21 (0.53)	0.48–2.91	0.29–2.39	0.745	1.22 (0.36)	0.41–2.03	0.35–3.88	0.92 (0.25)	0.34–1.49	0.03–2.39	0.597
	Oro-vestibular	0.49 (0.07)	0.35–0.63	0.10–0.98	0.88 (0.29)	0.42–0.80	0.20–1.58	0.769	0.45 (0.09)	0.24–0.65	0.10–0.98	0.69 (0.13)	0.40–0.98	0.17–1.58	0.495
	Apico-coronal	1.25 (0.23)	0.76–1.74	0.03–3.68	1.52 (0.68)	0.64–3.68	0.65–3.53	0.635	1.16 (0.35)	0.36–1.95	0 03–3 68	1.45 (0.28)	0.82–2.08	0.65–3.53	0.520
	Angular Deviation (degree)	2.70 (0.30)	2.10–3.40	1.20–5.90	2.70 (0.60)	0.70–4.60	1.10–4.00	0.905	2.90 (0.50)	1.9–3.90	1.20–5.90	2.50 (0.30)	1.90–3.10	1.10–4.00	0.273

Group comparison of the jaw and the surgical procedure with a *p* < 0.05. SD, standard deviation; CI, confidence interval.

## Data Availability

Not applicable.
